# Penta­fluoro­phenyl (3*R*,4*R*,5*S*)-5-{[(3*R*,4*R*,5*S*)-5-azido­methyl-3,4-dimeth­oxy-2,3,4,5-tetra­hydro­furan-3-carboxamido]­meth­yl}-3,4-dimeth­oxy-2,3,4,5-tetra­hydro­furan-3-carboxyl­ate

**DOI:** 10.1107/S1600536810038559

**Published:** 2010-10-02

**Authors:** Michela I. Simone, Alison A. Edwards, Samuel G. Parker, George E. Tranter, George W. J. Fleet, David J. Watkin

**Affiliations:** a Chemistry Research Laboratory, University of Oxford, Mansfield Road, Oxford OX1 3TA, England; b Medway School of Pharmacy, Universities of Kent and Greenwich at Medway, Central Avenue, Chatham Maritime, Kent ME4 4TB, England; c Chiralabs Limited, Begbroke Centre for Innovation and Enterprise, Oxford University Begbroke Science Park, Oxfordshire, England

## Abstract

The title compound, C_22_H_25_F_5_N_4_O_9_, is a stable penta­fluoro­phenyl ester inter­mediate in the synthesis of novel homo-oligomeric structures containing branched carbon chains. The structure is epimeric to the previously characterized dimeric penta­fluoro­phenyl ester with stereochemistry (3*R*,4*R*,5*R*), which was synthesized using d-ribose as starting material. The crystal structure of the title mol­ecule removes any ambiguities arising from the relative stereochemistries of the six chiral centres. Two hydrogen bonds, bifurcating from the NH group, stabilize the crystal: one intra­molecular and one inter­molecular, both involving O atoms of the meth­oxy groups. The asymmetric unit contains two independent mol­ecules not related by any pseudo-symmetry operators. The major conformational differences are localized, leading to one mol­ecule being extended compared to the other. The collected crystal was twinned (twin ratio is 0.939:0.061), and the azide group is positionally disordered over two positions in one mol­ecule [occupancy ratio 0.511 (18):0.489 (18)].

## Related literature

For the synthesis and use of sugar amino acids, see: Smith & Fleet (1999 ▶); Gibson *et al.* (2009 ▶); Mayes, Stetz *et al.* (2004 ▶); Hungerford *et al.* (2000 ▶); Jagadeesh *et al.* (2009 ▶); Risseeuw *et al.* (2007 ▶); Edwards *et al.* (2008 ▶). For the synthesis of penta­fluoro­phenyl esters in this series of compounds, see: Mayes, Cowley *et al.* (2004 ▶); Mayes, Simon *et al.* (2004 ▶). For other procedures for the synthesis of branched sugars, see: Ho & Wong (1985 ▶); Simone *et al.* (2005 ▶). For the synthesis of the title compound, see: Simone *et al.* (2008 ▶, 2010 ▶). For structures related to the title mol­ecule, and their characteristic features, see: Punzo *et al.* (2006 ▶); Humphreys *et al.* (2005 ▶).
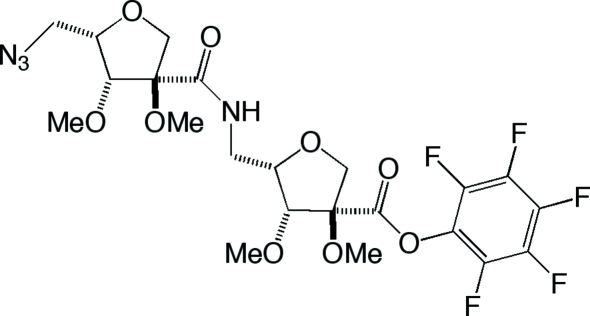


## Experimental

### 

#### Crystal data

C_22_H_25_F_5_N_4_O_9_
*M* *_r_* = 584.45
Monoclinic, 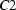

*a* = 26.8973 (4) Å
*b* = 7.9070 (1) Å
*c* = 24.7763 (5) Å
β = 102.4436 (6)°
*V* = 5145.56 (15) Å^3^
*Z* = 8
Mo *K*α radiation
μ = 0.14 mm^−1^
*T* = 150 K
0.80 × 0.08 × 0.08 mm

#### Data collection

Nonius KappaCCD diffractometer
Absorption correction: multi-scan (*DENZO*/*SCALEPACK*; Otwinowski & Minor, 1997 ▶) *T*_min_ = 0.76, *T*_max_ = 0.99
17175 measured reflections
4754 independent reflections
4160 reflections with *I* > 2.0σ(*I*)
*R*_int_ = 0.023

#### Refinement

*R*[*F* ^2^ > 2σ(*F* ^2^)] = 0.057
*wR*(*F* ^2^) = 0.159
*S* = 0.99
4754 reflections
759 parameters
83 restraints
H-atom parameters constrained
Δρ_max_ = 0.90 e Å^−3^
Δρ_min_ = −0.43 e Å^−3^

### 

Data collection: *COLLECT* (Nonius, 2001 ▶); cell refinement: *DENZO*/*SCALEPACK* (Otwinowski & Minor, 1997 ▶); data reduction: *DENZO*/*SCALEPACK*; program(s) used to solve structure: *SIR92* (Altomare *et al.*, 1994 ▶); program(s) used to refine structure: *CRYSTALS* (Betteridge *et al.*, 2003 ▶; Cooper *et al.*, 2010 ▶); molecular graphics: *CAMERON* (Watkin *et al.*, 1996 ▶); software used to prepare material for publication: *CRYSTALS*.

## Supplementary Material

Crystal structure: contains datablocks global, I. DOI: 10.1107/S1600536810038559/bh2308sup1.cif

Structure factors: contains datablocks I. DOI: 10.1107/S1600536810038559/bh2308Isup2.hkl

Additional supplementary materials:  crystallographic information; 3D view; checkCIF report

## Figures and Tables

**Table 1:** Hydrogen-bond geometry (Å, °)

*D*—H⋯*A*	*D*—H	H⋯*A*	*D*⋯*A*	*D*—H⋯*A*
C5—H52⋯O27^i^	0.97	2.23	3.131 (10)	153
C20—H202⋯N222^ii^	0.97	2.32	3.269 (10)	167
C26—H263⋯N222^ii^	0.97	2.51	3.214 (10)	129
C29—H293⋯O27^i^	0.97	2.49	3.278 (10)	139
N12—H121⋯O128^iii^	0.87	2.36	3.200 (10)	164
C129—H1292⋯O127^i^	0.96	2.46	3.370 (10)	158
C126—H1261⋯O27	0.96	2.47	3.202 (10)	133
N112—H1121⋯O28	0.86	2.33	3.147 (10)	159

Symmetry codes: (i) 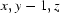; (ii) 
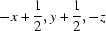
; (iii) 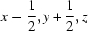.

**Table 2:** Selected torsion angles (°) leading to extended and contracted geometries.

Atoms	mol­ecule *A*	mol­ecule *B*
O6—C7—C11—N12	71.6	104.2
C7—C11—N12—C13	78.1	−176.8
C11—N12—C13—C14	−165.8	−174.1
N12—C13—C14—O25	−18.6	−155.9

**Fig. 1.**
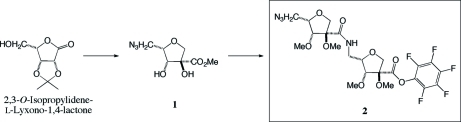
Synthetic route for the title compound (2).

**Fig. 2.**
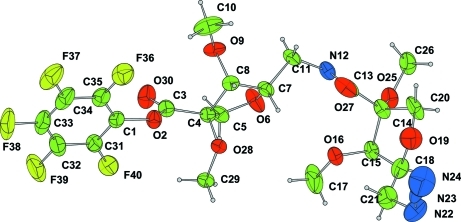
The contracted molecule (A) in the title compound with displacement ellipsoids drawn at the 50% probability level. H atoms are shown as spheres of arbitrary radius.

**Fig. 3.**
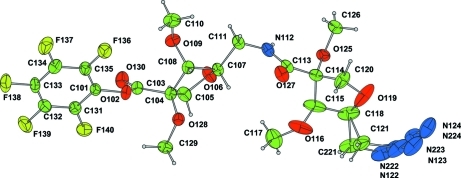
The extended molecule (B) in the title compound with displacement ellipsoids drawn at the 50% probability level. H atoms are shown as spheres of arbitrary radius.

**Fig. 4.**
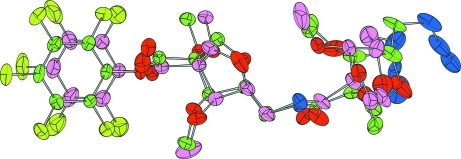
Best-match projection of molecule A and B. The C atoms in molecule A are red, those in B green.

**Fig. 5.**
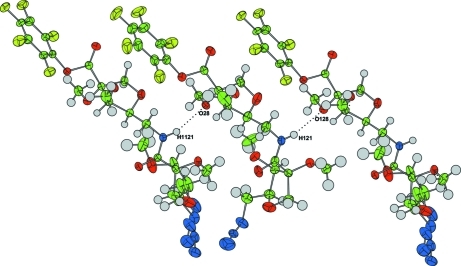
Part of the hydrogen bond ribbon. Even though molecules A and B have different configurations, the hydrogen bonding between A and B is very similar to that between B and A (Table 1). The short internal contact involving N—H···O is probably not a real hydrogen bond because the angle is too acute, but it may play a role in conserving the geometry in this region (Table 1).

**Fig. 6.**
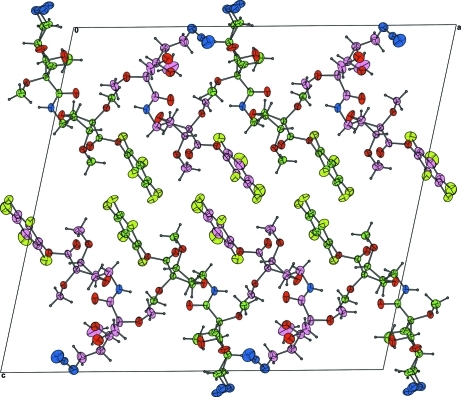
The hydrogen bonded ribbons are packed side-by-side into layers. The aromatic face of one layer lies adjacent to the corresponding face of an adjacent layer.

## supplementary crystallographic information

### Comment

Sugar amino acids (SAAs) have been extensively used in the design of peptidomimetics (Smith & Fleet, 1999; Gibson *et al.*, 2009), cyclodextrin mimics (Mayes, Stetz *et al.*, 2004), and foldamers (Hungerford *et al.*, 2000; Jagadeesh *et al.*, 2009). Foldamers provide increased understanding of the factors which induce secondary structures in proteins (Edwards *et al.*, 2008). Pentafluorophenyl esters have been shown to be particularly useful in the synthesis of homo-oligomers of SAAs (Mayes, Cowley *et al.*, 2004; Mayes, Simon *et al.*, 2004). Hitherto, all SAAs (Risseeuw *et al.*, 2007) used as peptidomimetics contain linear carbon chains. Efficient syntheses of branched sugars by the Ho-crossed aldol condensation (Ho & Wong, 1985; Simone *et al.*, 2005) allows access to carbon-branched SAA scaffolds, such as (1), which may provide monomers for new classes of foldamers.

The key step in the synthesis of the branched SAA precursor (1) is the reaction of formaldehyde with a suitably protected lactol derived from 2,3-*O*-isopropylidene-*L*-lyxono-1,4-lactone (Simone *et al.*, 2008). The SAA precursor (1) was converted by standard peptide procedures into the branched dimeric pentafluorophenyl ester (2)- a key intermediate in the synthesis of longer homo-oligomeric carbopeptoids (Simone *et al.*, 2010). The absolute configuration of the dimeric branched pentafluorophenyl ester (2) was determined by the use of 2,3-*O*-isopropylidene-*L*-lyxono-1,4-lactone as the starting material (Fig. 1). This structure is epimeric to the dimeric pentafluorophenyl ester with stereochemistry (3*R*,4*R*,5*R*) (Punzo *et al.*, 2006) which was synthesized using D-ribose as starting material.

The title material (2) crystallizes with two molecules in the asymmetric unit (*Z*'= 2, Fig. 2 and 3), and is twinned (twin law 1,0,0/0,-1,0/-0.397,0,-1; twin ratio 0.939:0.061). The two molecules have substantially different conformations (r.m.s. positional deviations after best-matching = 1.07 Å, r.m.s. torsion angle deviation = 22.3°). However, the local geometries in both are quite normal (r.m.s. bond length deviation = 0.02 Å). The principal differences are in the region O6—C7—C11—N12—C13—C14—O25 (in molecule *A*, Fig. 2) and the corresponding region O106—C107—C111—N112—C113—C114—O125 (in molecule *B*, Fig. 3; see Table 2). The especially large deviations for C7—C11—N12—C13 and N12—C13—C14—O25 leads to molecule *A* being partially folded back on itself so that it is less extended than molecule *B* (Fig. 4). Molecule *B* has disorder in the azide that can be modelled as two distinct sites. In both *A* and *B*, some of the atoms in the 5-membered rings and adjacent methyl groups show large adp's. This is consistent with the ring fluxion commonly seen in this class of compounds, and cannot really be modelled as split atoms. The non-linearity of the azide group [N22—N23—N24: 170.6 (8)°] and slight alternation in the N adp's are common in this class of structures (Humphreys *et al.*, 2005).

The crystal structure consists of infinite chains with an equivalent hydrogen bond linking molecule *A* to *B* as that linking *B* to the next *A* (Fig. 5). These chains are stacked side-by-side to form layers (Fig. 6). One face of this layer consists of pentafluorophenyl groups, the other face contains the terminal azide groups. The aromatic face is essentially flat, and opposes the aromatic face of the adjacent layer. The azide face is pleated (as a result of the differing over-all length of the molecules), with the ridges in one layer fitting into the hollows of the next.

### Experimental

The title compound (2) was obtained (Simone *et al.*, 2010) as a colourless oil which crystallized on standing and was recrystallized from ethyl acetate/petroleum ether 60–80°C. *M*.p. 364.2–365.2 K; m/*z* (ES^+^): 585.2 ([*M*+H]^+^, 60%), 607.2 ([*M*+H]^+^, 37%), 1191.1 ([2*M*+Na]^+^, 100%); HRMS (ES^+^): found 585.1612 [*M*+H]^+^ C_22_H_26_N_4_O_9_F_5_ requires 585.1620; ν_max_ (thin film): 3426 (br s, NH), 2947, 2839 (C—H), 2103 (s, N_3_), 1784 (m, C=OOPfp), 1657 (s, C=ONH, I), 1524 (s, C=ONH, II) cm^-1^; [α]_D_^24^ -32.5 (c, 0.17 in dichloromethane); δ_H_ (C_6_D_6_, 500 MHz): 3.00 (3*H*, s, C^3^OCH_3_, A), 3.18 (3*H*, s, C^3^OCH_3_, B), 3.26 (3*H*, s, C^4^OCH_3_, B), 3.30 (1*H*, dd, *J*_H-6,H-6'_ 12.6 Hz, *J*_H-6,H-5_ 6.3 Hz, H-6, A), 3.33 (3*H*, s, C^4^OCH_3_, A), 3.42 (1*H*, ddd, *J*_H-6,H-6'_ 13.1 Hz, *J*_H-6,H-5_ 8.5 Hz, *J*_H-6,NH_ 4.0 Hz, H-6, B), 3.53 (1*H*, dd, *J*_H-6',H-6_ 12.7 Hz, *J*_H-6',H-5_ 7.2 Hz, H-6', A), 3.58 (1*H*, d, *J*_H-4,H-5_ 4.8 Hz, H-4, A), 3.85 (1*H*, d, *J*_H-4,H-5_ 4.3 Hz, H-4, B), 4.02–4.05 (1*H*, m, H-6', B), 4.06 (1*H*, d, *J*_H-2,H-2'_ 10.5 Hz, H-2, B), 4.09 (1*H*, d, *J*_H-2,H-2'_ 10.7 Hz, H-2, A), 4.32–4.38 (1*H*, m, H-5, A), 4.34–4.40 (1*H*, m, H-5, B), 4.53 (1*H*, d, *J*_H-2',H-2_ 10.4 Hz, H-2', B), 4.77 (1*H*, d, *J*_H-2',H-2_ 10.7 Hz, H-2', A), 6.85–6.90 (1*H*, m, *J*_NH,H-6_ 4.0 Hz, NH); δ_C_ (C_6_D_6_, 125 MHz): 38.8 (C-6, B), 49.9 (C-6, A), 52.0 (C^3^OCH_3_, A), 53.1 (C^3^OCH_3_, B), 59.9 (C^4^OCH_3_, A), 60.5 (C^4^OCH_3_, B), 67.4 (C-2, A), 68.6 (C-2, B), 79.9 (C-5, B), 80.3 (C-5, A), 88.1 (C-4, B), 88.3 (C-4, A), 90.5 (C-3, B), 91.7 (C-3, A), 124.8 (m, ArCq), 137.1, 139.1 (2 C, m, 2 *x meta* Ar—CH), 138.7, 140.7 (1 C, m, *para* Ar—CH), 140.2, 142.1 (2 C, m, 2 *x ortho* Ar—CH), 165.2 (CONH), 168.2 (CO_2_Pfp); δ_F_ (C_6_D_6_, 376 MHz): -162.3 (2 F, dd, ^3^*J*_F_*_meta_*_,F_*_para_* 23.2 Hz, ^3^*J*_F_*_meta_*_,F_*_ortho_* 18.8 Hz, 2 *x* F*_meta_*_)_, -157.3 (1 F, t, ^3^*J*_F_*_para_*_,F_*_meta_* 23.3 Hz, F*_para_*), -153.0 (2 F, d, ^3^*J*_F_*_ortho_*_,F_*_meta_* 18.5 Hz, 2 *x* F*_ortho_*).

### Refinement

In the absence of significant anomalous scattering, Friedel pairs were merged and the absolute configuration assigned from the known starting materials.

The relatively large ratio of minimum to maximum corrections applied in the multiscan process (1:1.30) reflects changes in the illuminated volume of the crystal. These were kept to a minimum, and were taken into account by the multi-scan inter-frame scaling (*DENZO*/*SCALEPACK*, Otwinowski & Minor, 1997).

The H atoms were all located in a difference map, but those attached to C atoms were repositioned geometrically. All H atoms except that on C118 (at the start of the disordered azide group) were initially refined with soft restraints on the bond lengths and angles to regularize their geometry (C—H in the range 0.93–0.98, N—H to 0.86 Å) and *U*_iso_(H) (in the range 1.2–1.5 times *U*_eq_ of the parent atom), after which the positions were refined with riding constraints (Cooper *et al.*, 2010).

In spite of the poor quality of the crystal, distance and adp similarity restraints were only necessary for the disordered azide group in molecule *B*.

### Crystal data

**Table:**

C_22_H_25_F_5_N_4_O_9_	*F*(000) = 2416.000
*M**_r_* = 584.45	*D*_x_ = 1.509 Mg m^−^^3^
Monoclinic, *C*2	Melting point: 364.2 K
Hall symbol: C 2y	Mo *K*α radiation, λ = 0.71073 Å
*a* = 26.8973 (4) Å	Cell parameters from 4194 reflections
*b* = 7.9070 (1) Å	θ = 5–25°
*c* = 24.7763 (5) Å	µ = 0.14 mm^−^^1^
β = 102.4436 (6)°	*T* = 150 K
*V* = 5145.56 (15) Å^3^	Plate, colourless
*Z* = 8	0.80 × 0.08 × 0.08 mm

### Data collection

**Table:**

Nonius KappaCCD diffractometer	4160 reflections with *I* > 2.0σ(*I*)
graphite	*R*_int_ = 0.023
ω scans	θ_max_ = 25.0°, θ_min_ = 5.1°
Absorption correction: multi-scan (*DENZO*/*SCALEPACK*; Otwinowski & Minor, 1997)	*h* = −31→32
*T*_min_ = 0.76, *T*_max_ = 0.99	*k* = −8→9
17175 measured reflections	*l* = −29→29
4754 independent reflections	

### Refinement

**Table:**

Refinement on *F*^2^	0 constraints
Least-squares matrix: full	Primary atom site location: structure-invariant direct methods
*R*[*F*^2^ > 2σ(*F*^2^)] = 0.057	Hydrogen site location: inferred from neighbouring sites
*wR*(*F*^2^) = 0.159	H-atom parameters constrained
*S* = 0.99	*w* = 1/[σ^2^(*F*^2^) + (0.12*P*)^2^ + 4.59*P*], where *P* = [max(*F*_o_^2^,0) + 2*F*_c_^2^]/3
4754 reflections	(Δ/σ)_max_ = 0.0003
759 parameters	Δρ_max_ = 0.90 e Å^−^^3^
83 restraints	Δρ_min_ = −0.43 e Å^−^^3^

### Special details

**Table:**

Experimental. The crystal was placed in the cold stream of an Oxford Cryosystems open-flow nitrogen cryostat, with a nominal stability of 0.1 K.

### Fractional atomic coordinates and isotropic or equivalent isotropic displacement parameters (Å^2^)

**Table:**

	*x*	*y*	*z*	*U*_iso_*/*U*_eq_	Occ. (<1)
C1	0.4720 (2)	0.0750 (9)	0.3805 (2)	0.0420	
O2	0.44018 (13)	0.1870 (6)	0.34614 (16)	0.0397	
C3	0.38899 (19)	0.1640 (8)	0.3421 (2)	0.0393	
C4	0.35842 (18)	0.2805 (7)	0.2970 (2)	0.0334	
C5	0.30354 (19)	0.2195 (8)	0.2792 (3)	0.0406	
O6	0.27353 (15)	0.3616 (6)	0.2641 (2)	0.0581	
C7	0.3045 (2)	0.5121 (7)	0.2749 (2)	0.0386	
C8	0.35104 (18)	0.4609 (7)	0.3173 (2)	0.0334	
O9	0.34056 (13)	0.4538 (6)	0.37104 (15)	0.0449	
C10	0.3731 (3)	0.5595 (13)	0.4103 (3)	0.0746	
C11	0.2724 (2)	0.6557 (8)	0.2890 (3)	0.0440	
N12	0.23588 (15)	0.7098 (6)	0.2389 (2)	0.0377	
C13	0.2519 (2)	0.8066 (7)	0.2025 (3)	0.0418	
C14	0.21709 (19)	0.8268 (7)	0.1449 (3)	0.0408	
C15	0.2357 (2)	0.7039 (8)	0.1041 (2)	0.0396	
O16	0.27759 (15)	0.6052 (6)	0.13194 (17)	0.0465	
C17	0.2760 (5)	0.4357 (10)	0.1194 (5)	0.1005	
C18	0.2499 (2)	0.8238 (9)	0.0613 (3)	0.0500	
O19	0.2628 (2)	0.9862 (7)	0.0925 (2)	0.0662	
C20	0.2237 (3)	1.0042 (8)	0.1210 (4)	0.0604	
C21	0.2956 (3)	0.7817 (11)	0.0418 (3)	0.0663	
N22	0.3103 (2)	0.9143 (9)	0.0060 (2)	0.0644	
N23	0.33914 (17)	1.0349 (8)	0.0317 (2)	0.0455	
N24	0.3598 (3)	1.1349 (12)	0.0451 (4)	0.0996	
O25	0.16578 (14)	0.7812 (6)	0.14434 (18)	0.0475	
C26	0.1388 (3)	0.9057 (11)	0.1697 (3)	0.0646	
O27	0.29382 (14)	0.8802 (6)	0.2127 (2)	0.0511	
O28	0.38321 (12)	0.3016 (5)	0.25249 (14)	0.0301	
C29	0.38704 (19)	0.1522 (8)	0.2206 (2)	0.0367	
O30	0.37183 (15)	0.0637 (7)	0.3697 (2)	0.0587	
C31	0.4741 (3)	−0.0927 (9)	0.3682 (3)	0.0514	
C32	0.5094 (3)	−0.1973 (12)	0.4007 (4)	0.0747	
C33	0.5434 (3)	−0.1318 (14)	0.4461 (3)	0.0724	
C34	0.5418 (2)	0.0377 (14)	0.4584 (3)	0.0654	
C35	0.5070 (2)	0.1392 (11)	0.4262 (3)	0.0514	
F36	0.50605 (14)	0.3058 (7)	0.43773 (17)	0.0658	
F37	0.57451 (14)	0.1043 (9)	0.50139 (17)	0.0893	
F38	0.5772 (2)	−0.2327 (9)	0.4771 (2)	0.1124	
F39	0.5108 (3)	−0.3608 (7)	0.3887 (3)	0.1051	
F40	0.44264 (17)	−0.1554 (6)	0.32414 (17)	0.0674	
H51	0.2930	0.1663	0.3101	0.0515*	
H52	0.2992	0.1399	0.2486	0.0513*	
H71	0.3164	0.5395	0.2409	0.0450*	
H81	0.3792	0.5329	0.3159	0.0414*	
H101	0.3618	0.5613	0.4445	0.1108*	
H102	0.4076	0.5171	0.4164	0.1109*	
H103	0.3719	0.6722	0.3955	0.1114*	
H111	0.2539	0.6184	0.3168	0.0536*	
H112	0.2937	0.7512	0.3025	0.0536*	
H151	0.2073	0.6292	0.0873	0.0484*	
H171	0.3063	0.3811	0.1416	0.1369*	
H172	0.2756	0.4218	0.0805	0.1373*	
H173	0.2452	0.3864	0.1274	0.1370*	
H181	0.2193	0.8517	0.0330	0.0616*	
H201	0.2328	1.0904	0.1501	0.0843*	
H202	0.1922	1.0373	0.0961	0.0842*	
H211	0.2902	0.6730	0.0219	0.0866*	
H212	0.3233	0.7699	0.0747	0.0861*	
H261	0.1043	0.8660	0.1674	0.1063*	
H262	0.1557	0.9180	0.2081	0.1062*	
H263	0.1385	1.0131	0.1508	0.1062*	
H291	0.4064	0.1752	0.1929	0.0577*	
H292	0.4042	0.0641	0.2445	0.0578*	
H293	0.3534	0.1133	0.2030	0.0571*	
H121	0.2049	0.6703	0.2314	0.0453*	
C117	0.5021 (3)	0.2319 (13)	0.0929 (4)	0.0801	
O116	0.49194 (18)	0.4042 (8)	0.0831 (2)	0.0722	
C115	0.4454 (2)	0.4581 (10)	0.0950 (3)	0.0550	
C114	0.45486 (17)	0.5879 (7)	0.1427 (2)	0.0310	
C120	0.4646 (3)	0.7465 (9)	0.1117 (2)	0.0497	
O119	0.4268 (2)	0.7415 (9)	0.06111 (19)	0.0794	
C118	0.4153 (2)	0.5647 (11)	0.0463 (2)	0.0621	
C121	0.4308 (5)	0.575 (2)	−0.0090 (4)	0.0581	0.511 (18)
N122	0.3979 (5)	0.6849 (15)	−0.0521 (5)	0.0483	0.511 (18)
N123	0.4064 (5)	0.8486 (15)	−0.0497 (5)	0.0443	0.511 (18)
N124	0.4131 (5)	0.9747 (17)	−0.0512 (5)	0.0637	0.511 (18)
C113	0.49815 (17)	0.5442 (7)	0.1907 (2)	0.0312	
N112	0.48531 (14)	0.4574 (6)	0.23224 (18)	0.0337	
C111	0.5243 (2)	0.3948 (8)	0.2780 (2)	0.0373	
C107	0.55076 (18)	0.2409 (7)	0.2601 (2)	0.0312	
O106	0.51648 (13)	0.0987 (5)	0.25502 (17)	0.0412	
C105	0.54569 (18)	−0.0488 (8)	0.2533 (2)	0.0372	
C104	0.60205 (17)	−0.0040 (7)	0.2762 (2)	0.0291	
C108	0.59836 (18)	0.1781 (7)	0.2994 (2)	0.0320	
O109	0.59212 (13)	0.1648 (5)	0.35444 (15)	0.0383	
C110	0.6201 (2)	0.2895 (9)	0.3900 (2)	0.0492	
C103	0.62809 (17)	−0.1243 (7)	0.3212 (2)	0.0308	
O102	0.67965 (12)	−0.0920 (5)	0.33282 (14)	0.0312	
C101	0.71065 (17)	−0.1939 (7)	0.3717 (2)	0.0301	
C131	0.71597 (19)	−0.3638 (7)	0.3636 (2)	0.0333	
C132	0.7505 (2)	−0.4588 (8)	0.4010 (2)	0.0419	
C133	0.7793 (2)	−0.3825 (8)	0.4467 (2)	0.0398	
C134	0.7739 (2)	−0.2145 (8)	0.4554 (2)	0.0397	
C135	0.73976 (19)	−0.1191 (7)	0.4179 (2)	0.0337	
F136	0.73630 (13)	0.0473 (5)	0.42632 (14)	0.0484	
F137	0.80219 (14)	−0.1370 (6)	0.50043 (14)	0.0575	
F138	0.81310 (13)	−0.4746 (5)	0.48326 (15)	0.0551	
F139	0.75578 (15)	−0.6241 (5)	0.39232 (16)	0.0559	
F140	0.68948 (12)	−0.4392 (5)	0.31769 (13)	0.0459	
O130	0.60865 (14)	−0.2288 (5)	0.34484 (17)	0.0434	
O128	0.63024 (12)	0.0180 (5)	0.23410 (14)	0.0319	
C129	0.63836 (19)	−0.1350 (8)	0.2052 (2)	0.0382	
O127	0.54240 (12)	0.5832 (5)	0.19084 (17)	0.0420	
O125	0.40702 (12)	0.5920 (5)	0.15871 (14)	0.0350	
C126	0.3985 (2)	0.7382 (9)	0.1890 (3)	0.0533	
C221	0.4201 (5)	0.4784 (19)	−0.0043 (4)	0.0622	0.489 (18)
N222	0.3914 (4)	0.581 (2)	−0.0501 (4)	0.0713	0.489 (18)
N223	0.4109 (6)	0.730 (3)	−0.0551 (7)	0.0682	0.489 (18)
N224	0.4213 (7)	0.848 (2)	−0.0642 (7)	0.0742	0.489 (18)
H1171	0.5378	0.2119	0.0944	0.1160*	
H1172	0.4817	0.1663	0.0638	0.1159*	
H1173	0.4944	0.2023	0.1280	0.1160*	
H1151	0.4263	0.3604	0.1032	0.0717*	
H1201	0.4993	0.7468	0.1062	0.0583*	
H1202	0.4591	0.8459	0.1319	0.0583*	
H1111	0.5084	0.3635	0.3083	0.0432*	
H1112	0.5502	0.4822	0.2899	0.0435*	
H1071	0.5596	0.2633	0.2239	0.0365*	
H1051	0.5413	−0.0905	0.2154	0.0461*	
H1052	0.5354	−0.1370	0.2760	0.0454*	
H1081	0.6282	0.2477	0.2973	0.0372*	
H1101	0.6117	0.2792	0.4257	0.0722*	
H1102	0.6113	0.4009	0.3753	0.0721*	
H1103	0.6556	0.2705	0.3940	0.0721*	
H1291	0.6531	−0.1053	0.1746	0.0582*	
H1292	0.6069	−0.1947	0.1926	0.0584*	
H1293	0.6617	−0.2071	0.2299	0.0583*	
H1261	0.3708	0.7169	0.2070	0.0804*	
H1262	0.3899	0.8332	0.1646	0.0800*	
H1263	0.4291	0.7634	0.2163	0.0800*	
H1121	0.4538	0.4325	0.2307	0.0413*	
H1211	0.4310	0.4576	−0.0243	0.0720*	0.511 (18)
H1212	0.4661	0.6221	−0.0016	0.0720*	0.511 (18)
H2211	0.4058	0.3615	−0.0054	0.0681*	0.489 (18)
H2212	0.4568	0.4724	−0.0061	0.0681*	0.489 (18)
H1181	0.3782	0.5463	0.0443	0.0747*	

### Atomic displacement parameters (Å^2^)

**Table:**

	*U*^11^	*U*^22^	*U*^33^	*U*^12^	*U*^13^	*U*^23^
C1	0.037 (3)	0.055 (4)	0.037 (3)	0.011 (3)	0.016 (2)	0.012 (3)
O2	0.0326 (17)	0.043 (2)	0.044 (2)	0.0024 (17)	0.0089 (16)	0.0094 (18)
C3	0.033 (3)	0.044 (3)	0.045 (3)	0.004 (2)	0.018 (2)	0.009 (3)
C4	0.031 (2)	0.036 (3)	0.035 (3)	0.001 (2)	0.011 (2)	0.002 (2)
C5	0.032 (3)	0.038 (3)	0.056 (4)	0.001 (2)	0.019 (2)	−0.002 (3)
O6	0.038 (2)	0.032 (2)	0.090 (3)	0.0027 (18)	−0.017 (2)	−0.020 (2)
C7	0.038 (3)	0.033 (3)	0.042 (3)	0.005 (2)	0.002 (2)	−0.010 (2)
C8	0.030 (2)	0.037 (3)	0.033 (3)	−0.006 (2)	0.007 (2)	−0.005 (2)
O9	0.0368 (18)	0.063 (3)	0.037 (2)	−0.0122 (19)	0.0117 (16)	−0.017 (2)
C10	0.074 (4)	0.103 (7)	0.045 (4)	−0.036 (5)	0.010 (3)	−0.032 (4)
C11	0.046 (3)	0.033 (3)	0.053 (3)	0.008 (3)	0.010 (3)	−0.012 (3)
N12	0.025 (2)	0.032 (2)	0.055 (3)	0.0005 (19)	0.0082 (19)	0.001 (2)
C13	0.034 (3)	0.027 (3)	0.065 (4)	0.004 (2)	0.012 (2)	−0.010 (3)
C14	0.033 (3)	0.029 (3)	0.064 (4)	0.001 (2)	0.020 (2)	0.008 (3)
C15	0.039 (3)	0.029 (3)	0.050 (3)	0.002 (2)	0.010 (2)	0.007 (3)
O16	0.051 (2)	0.037 (2)	0.050 (2)	0.0119 (19)	0.0072 (18)	0.003 (2)
C17	0.144 (9)	0.030 (4)	0.103 (7)	0.020 (5)	−0.028 (6)	−0.004 (4)
C18	0.052 (3)	0.051 (4)	0.046 (3)	−0.005 (3)	0.008 (3)	0.009 (3)
O19	0.081 (3)	0.047 (3)	0.075 (3)	−0.013 (3)	0.026 (3)	0.002 (3)
C20	0.059 (4)	0.032 (3)	0.103 (6)	0.015 (3)	0.043 (4)	0.023 (3)
C21	0.063 (4)	0.065 (5)	0.078 (5)	0.003 (4)	0.031 (4)	0.020 (4)
N22	0.061 (3)	0.079 (4)	0.055 (3)	−0.023 (3)	0.016 (3)	0.010 (3)
N23	0.037 (2)	0.060 (3)	0.039 (2)	−0.0045 (19)	0.0064 (19)	0.023 (2)
N24	0.101 (5)	0.078 (5)	0.112 (5)	−0.033 (4)	0.006 (4)	0.004 (4)
O25	0.0341 (18)	0.047 (2)	0.062 (2)	0.0003 (18)	0.0126 (17)	0.012 (2)
C26	0.057 (4)	0.072 (5)	0.076 (5)	0.026 (4)	0.038 (4)	0.035 (4)
O27	0.0330 (19)	0.036 (2)	0.086 (3)	−0.0095 (18)	0.0182 (19)	−0.017 (2)
O28	0.0293 (15)	0.0281 (18)	0.0354 (18)	−0.0036 (14)	0.0122 (14)	−0.0013 (15)
C29	0.035 (2)	0.039 (3)	0.039 (3)	0.001 (2)	0.013 (2)	−0.002 (2)
O30	0.042 (2)	0.070 (3)	0.068 (3)	0.002 (2)	0.022 (2)	0.030 (3)
C31	0.062 (4)	0.051 (4)	0.047 (4)	0.018 (3)	0.026 (3)	0.015 (3)
C32	0.085 (5)	0.071 (5)	0.081 (6)	0.040 (5)	0.046 (5)	0.037 (5)
C33	0.061 (4)	0.101 (7)	0.062 (5)	0.031 (5)	0.027 (4)	0.045 (5)
C34	0.037 (3)	0.116 (8)	0.043 (4)	0.010 (4)	0.007 (3)	0.025 (4)
C35	0.038 (3)	0.075 (5)	0.045 (3)	0.008 (3)	0.016 (3)	0.013 (3)
F36	0.050 (2)	0.086 (3)	0.057 (2)	−0.001 (2)	0.0006 (17)	−0.003 (2)
F37	0.046 (2)	0.165 (6)	0.053 (2)	0.012 (3)	0.0010 (18)	0.024 (3)
F38	0.088 (3)	0.148 (6)	0.105 (4)	0.067 (4)	0.029 (3)	0.081 (4)
F39	0.134 (5)	0.063 (3)	0.133 (5)	0.051 (3)	0.062 (4)	0.043 (3)
F40	0.088 (3)	0.055 (3)	0.060 (2)	0.011 (2)	0.018 (2)	0.001 (2)
C117	0.064 (5)	0.088 (7)	0.085 (6)	0.031 (5)	0.010 (4)	0.006 (5)
O116	0.051 (3)	0.076 (4)	0.100 (4)	−0.007 (3)	0.039 (3)	−0.044 (3)
C115	0.034 (3)	0.062 (4)	0.075 (5)	−0.017 (3)	0.028 (3)	−0.039 (4)
C114	0.028 (2)	0.029 (3)	0.038 (3)	−0.004 (2)	0.013 (2)	−0.005 (2)
C120	0.059 (4)	0.050 (4)	0.034 (3)	−0.021 (3)	−0.003 (3)	0.005 (3)
O119	0.081 (3)	0.110 (5)	0.037 (2)	−0.046 (4)	−0.011 (2)	0.025 (3)
C118	0.039 (3)	0.101 (5)	0.047 (3)	−0.015 (3)	0.011 (2)	−0.028 (4)
C121	0.048 (5)	0.088 (7)	0.034 (4)	0.007 (5)	−0.002 (4)	−0.005 (5)
N122	0.043 (6)	0.067 (6)	0.030 (5)	−0.003 (5)	−0.004 (4)	−0.013 (5)
N123	0.034 (5)	0.068 (6)	0.032 (5)	−0.005 (5)	0.008 (4)	−0.001 (5)
N124	0.056 (6)	0.077 (7)	0.052 (6)	−0.022 (6)	−0.001 (5)	0.020 (6)
C113	0.028 (2)	0.022 (2)	0.045 (3)	0.003 (2)	0.012 (2)	−0.001 (2)
N112	0.0247 (19)	0.029 (2)	0.048 (3)	0.0015 (18)	0.0104 (18)	0.002 (2)
C111	0.035 (3)	0.033 (3)	0.045 (3)	0.006 (2)	0.011 (2)	0.003 (2)
C107	0.028 (2)	0.029 (3)	0.038 (3)	0.003 (2)	0.007 (2)	0.003 (2)
O106	0.0254 (16)	0.033 (2)	0.062 (2)	0.0001 (16)	0.0021 (16)	−0.0063 (19)
C105	0.023 (2)	0.038 (3)	0.049 (3)	−0.002 (2)	0.002 (2)	−0.005 (3)
C104	0.024 (2)	0.031 (3)	0.032 (3)	−0.001 (2)	0.0060 (19)	0.003 (2)
C108	0.029 (2)	0.032 (3)	0.033 (3)	−0.001 (2)	0.004 (2)	0.001 (2)
O109	0.0365 (18)	0.045 (2)	0.0333 (19)	−0.0085 (17)	0.0083 (15)	−0.0040 (17)
C110	0.046 (3)	0.056 (4)	0.042 (3)	−0.008 (3)	0.002 (2)	−0.017 (3)
C103	0.027 (2)	0.031 (3)	0.033 (3)	−0.001 (2)	0.0053 (19)	0.002 (2)
O102	0.0243 (15)	0.035 (2)	0.0326 (18)	−0.0005 (14)	0.0020 (13)	0.0118 (15)
C101	0.026 (2)	0.034 (3)	0.032 (3)	0.007 (2)	0.011 (2)	0.008 (2)
C131	0.033 (2)	0.033 (3)	0.036 (3)	0.001 (2)	0.012 (2)	0.005 (2)
C132	0.049 (3)	0.037 (3)	0.043 (3)	0.010 (3)	0.016 (3)	0.002 (3)
C133	0.039 (3)	0.038 (3)	0.044 (3)	0.013 (2)	0.011 (2)	0.018 (3)
C134	0.037 (3)	0.046 (3)	0.034 (3)	−0.002 (3)	0.003 (2)	0.004 (3)
C135	0.037 (3)	0.027 (3)	0.036 (3)	−0.001 (2)	0.004 (2)	0.002 (2)
F136	0.060 (2)	0.037 (2)	0.0441 (18)	0.0018 (16)	0.0029 (15)	−0.0008 (15)
F137	0.063 (2)	0.059 (3)	0.0395 (19)	−0.0037 (19)	−0.0133 (16)	0.0017 (17)
F138	0.0474 (17)	0.059 (2)	0.056 (2)	0.0174 (17)	0.0050 (16)	0.0249 (18)
F139	0.068 (2)	0.0328 (19)	0.070 (2)	0.0132 (17)	0.0213 (19)	0.0058 (18)
F140	0.0512 (17)	0.042 (2)	0.0420 (17)	−0.0038 (15)	0.0045 (14)	−0.0093 (15)
O130	0.0372 (19)	0.042 (2)	0.052 (2)	−0.0032 (17)	0.0132 (17)	0.016 (2)
O128	0.0317 (16)	0.0315 (19)	0.0328 (18)	−0.0007 (15)	0.0075 (14)	0.0030 (15)
C129	0.034 (2)	0.040 (3)	0.041 (3)	−0.003 (2)	0.008 (2)	−0.010 (3)
O127	0.0288 (17)	0.035 (2)	0.066 (2)	−0.0019 (16)	0.0172 (16)	0.0063 (19)
O125	0.0276 (16)	0.041 (2)	0.0385 (19)	0.0041 (16)	0.0116 (14)	−0.0029 (17)
C126	0.056 (3)	0.055 (4)	0.049 (3)	0.023 (3)	0.013 (3)	−0.004 (3)
C221	0.042 (5)	0.097 (8)	0.046 (5)	−0.030 (5)	0.005 (4)	−0.034 (5)
N222	0.056 (6)	0.110 (9)	0.047 (5)	−0.036 (6)	0.009 (4)	−0.020 (5)
N223	0.054 (7)	0.115 (11)	0.033 (6)	−0.031 (7)	0.004 (5)	0.006 (7)
N224	0.075 (9)	0.101 (11)	0.040 (7)	−0.015 (8)	−0.001 (6)	0.009 (8)

### Geometric parameters (Å, °)

**Table:**

C1—O2	1.388 (7)	O116—C115	1.411 (7)
C1—C31	1.365 (10)	C115—C114	1.545 (8)
C1—C35	1.401 (10)	C115—C118	1.548 (11)
O2—C3	1.371 (6)	C115—H1151	0.973
C3—C4	1.542 (8)	C114—C120	1.523 (8)
C3—O30	1.202 (7)	C114—C113	1.514 (7)
C4—C5	1.525 (7)	C114—O125	1.426 (5)
C4—C8	1.540 (8)	C120—O119	1.434 (7)
C4—O28	1.416 (6)	C120—H1201	0.971
C5—O6	1.387 (7)	C120—H1202	0.960
C5—H51	0.966	O119—C118	1.461 (11)
C5—H52	0.973	C118—C121	1.519 (8)
O6—C7	1.444 (7)	C118—H1181	1.000
C7—C8	1.507 (7)	C118—C221	1.458 (8)
C7—C11	1.512 (8)	C118—H1181	1.000
C7—H71	0.986	C121—N122	1.508 (9)
C8—O9	1.420 (6)	C121—H1211	1.000
C8—H81	0.954	C121—H1212	1.000
O9—C10	1.429 (8)	N122—N123	1.313 (9)
C10—H101	0.959	N123—N124	1.015 (9)
C10—H102	0.968	C113—N112	1.343 (7)
C10—H103	0.962	C113—O127	1.229 (6)
C11—N12	1.471 (8)	N112—C111	1.455 (7)
C11—H111	0.979	N112—H1121	0.863
C11—H112	0.963	C111—C107	1.524 (7)
N12—C13	1.324 (8)	C111—H1111	0.971
N12—H121	0.870	C111—H1112	0.981
C13—C14	1.538 (9)	C107—O106	1.442 (7)
C13—O27	1.245 (7)	C107—C108	1.515 (7)
C14—C15	1.559 (8)	C107—H1071	0.992
C14—C20	1.546 (8)	O106—C105	1.412 (7)
C14—O25	1.424 (6)	C105—C104	1.541 (6)
C15—O16	1.422 (7)	C105—H1051	0.979
C15—C18	1.531 (9)	C105—H1052	0.973
C15—H151	0.983	C104—C108	1.562 (7)
O16—C17	1.374 (9)	C104—C103	1.518 (7)
C17—H171	0.980	C104—O128	1.425 (6)
C17—H172	0.969	C108—O109	1.414 (6)
C17—H173	0.972	C108—H1081	0.983
C18—O19	1.500 (9)	O109—C110	1.424 (7)
C18—C21	1.453 (9)	C110—H1101	0.963
C18—H181	0.985	C110—H1102	0.962
O19—C20	1.397 (8)	C110—H1103	0.952
C20—H201	0.984	C103—O102	1.378 (6)
C20—H202	0.970	C103—O130	1.196 (6)
C21—N22	1.482 (9)	O102—C101	1.387 (6)
C21—H211	0.986	C101—C131	1.370 (8)
C21—H212	0.982	C101—C135	1.374 (8)
N22—N23	1.305 (8)	C131—C132	1.385 (8)
N23—N24	0.983 (9)	C131—F140	1.344 (6)
O25—C26	1.446 (9)	C132—C133	1.366 (9)
C26—H261	0.968	C132—F139	1.337 (7)
C26—H262	0.967	C133—C134	1.359 (9)
C26—H263	0.969	C133—F138	1.350 (6)
O28—C29	1.438 (7)	C134—C135	1.382 (8)
C29—H291	0.962	C134—F137	1.354 (7)
C29—H292	0.965	C135—F136	1.339 (7)
C29—H293	0.967	O128—C129	1.446 (7)
C31—C32	1.379 (10)	C129—H1291	0.958
C31—F40	1.325 (9)	C129—H1292	0.961
C32—C33	1.388 (14)	C129—H1293	0.963
C32—F39	1.329 (12)	O125—C126	1.423 (8)
C33—C34	1.377 (14)	C126—H1261	0.963
C33—F38	1.323 (9)	C126—H1262	0.961
C34—C35	1.356 (10)	C126—H1263	0.967
C34—F37	1.335 (10)	C221—N222	1.474 (9)
C35—F36	1.350 (9)	C221—H2211	1.000
C117—O116	1.400 (12)	C221—H2212	1.000
C117—H1171	0.967	N222—N223	1.301 (9)
C117—H1172	0.958	N223—N224	1.014 (9)
C117—H1173	0.967		
			
O2—C1—C31	122.2 (6)	O116—C115—C114	110.7 (4)
O2—C1—C35	118.8 (6)	O116—C115—C118	109.9 (6)
C31—C1—C35	118.6 (6)	C114—C115—C118	101.9 (6)
C1—O2—C3	115.9 (4)	O116—C115—H1151	109.7
O2—C3—C4	110.1 (4)	C114—C115—H1151	111.9
O2—C3—O30	123.3 (5)	C118—C115—H1151	112.6
C4—C3—O30	126.6 (5)	C115—C114—C120	100.3 (5)
C3—C4—C5	110.6 (5)	C115—C114—C113	114.9 (5)
C3—C4—C8	113.7 (4)	C120—C114—C113	113.4 (4)
C5—C4—C8	101.7 (4)	C115—C114—O125	102.7 (4)
C3—C4—O28	111.6 (4)	C120—C114—O125	112.6 (5)
C5—C4—O28	113.7 (4)	C113—C114—O125	111.9 (4)
C8—C4—O28	105.1 (4)	C114—C120—O119	104.6 (5)
C4—C5—O6	107.0 (5)	C114—C120—H1201	110.3
C4—C5—H51	109.8	O119—C120—H1201	113.4
O6—C5—H51	108.3	C114—C120—H1202	110.4
C4—C5—H52	112.0	O119—C120—H1202	108.6
O6—C5—H52	110.4	H1201—C120—H1202	109.4
H51—C5—H52	109.2	C120—O119—C118	108.5 (5)
C5—O6—C7	109.8 (4)	C115—C118—O119	106.2 (5)
O6—C7—C8	105.5 (5)	C115—C118—C121	122.1 (6)
O6—C7—C11	109.0 (4)	O119—C118—C121	95.3 (8)
C8—C7—C11	118.0 (5)	C115—C118—H1181	108.0
O6—C7—H71	107.7	O119—C118—H1181	108.0
C8—C7—H71	106.3	C121—C118—H1181	115.2
C11—C7—H71	109.8	C115—C118—O119	106.2 (5)
C4—C8—C7	99.6 (4)	C115—C118—C221	106.6 (8)
C4—C8—O9	109.6 (4)	O119—C118—C221	127.6 (8)
C7—C8—O9	111.3 (4)	C115—C118—H1181	108.0
C4—C8—H81	112.4	O119—C118—H1181	108.0
C7—C8—H81	111.1	C221—C118—H1181	99.2
O9—C8—H81	112.2	C118—C121—N122	115.8 (7)
C8—O9—C10	113.5 (5)	C118—C121—H1211	108.7
O9—C10—H101	110.3	N122—C121—H1211	108.5
O9—C10—H102	109.3	C118—C121—H1212	106.7
H101—C10—H102	110.0	N122—C121—H1212	107.5
O9—C10—H103	108.5	H1211—C121—H1212	109.5
H101—C10—H103	109.5	C121—N122—N123	118.0 (8)
H102—C10—H103	109.3	N122—N123—N124	175.3 (16)
C7—C11—N12	109.5 (5)	C114—C113—N112	115.9 (4)
C7—C11—H111	109.8	C114—C113—O127	121.7 (5)
N12—C11—H111	109.5	N112—C113—O127	122.4 (5)
C7—C11—H112	110.0	C113—N112—C111	120.7 (4)
N12—C11—H112	107.8	C113—N112—H1121	119.7
H111—C11—H112	110.2	C111—N112—H1121	119.4
C11—N12—C13	119.1 (4)	N112—C111—C107	110.3 (4)
C11—N12—H121	120.7	N112—C111—H1111	109.1
C13—N12—H121	119.9	C107—C111—H1111	109.4
N12—C13—C14	118.0 (5)	N112—C111—H1112	109.9
N12—C13—O27	123.1 (6)	C107—C111—H1112	107.7
C14—C13—O27	118.9 (5)	H1111—C111—H1112	110.4
C13—C14—C15	108.7 (4)	C111—C107—O106	108.5 (4)
C13—C14—C20	110.6 (5)	C111—C107—C108	117.2 (5)
C15—C14—C20	103.8 (5)	O106—C107—C108	103.7 (4)
C13—C14—O25	112.2 (4)	C111—C107—H1071	109.9
C15—C14—O25	106.3 (5)	O106—C107—H1071	109.5
C20—C14—O25	114.6 (5)	C108—C107—H1071	107.8
C14—C15—O16	111.1 (5)	C107—O106—C105	107.3 (3)
C14—C15—C18	103.0 (5)	O106—C105—C104	108.0 (4)
O16—C15—C18	112.6 (5)	O106—C105—H1051	110.7
C14—C15—H151	108.8	C104—C105—H1051	109.6
O16—C15—H151	109.7	O106—C105—H1052	110.2
C18—C15—H151	111.4	C104—C105—H1052	109.4
C15—O16—C17	116.3 (6)	H1051—C105—H1052	108.9
O16—C17—H171	108.8	C105—C104—C108	102.2 (4)
O16—C17—H172	109.2	C105—C104—C103	112.8 (4)
H171—C17—H172	109.7	C108—C104—C103	111.3 (4)
O16—C17—H173	109.4	C105—C104—O128	113.3 (4)
H171—C17—H173	110.6	C108—C104—O128	104.1 (4)
H172—C17—H173	109.1	C103—C104—O128	112.3 (4)
C15—C18—O19	103.8 (5)	C104—C108—C107	100.2 (4)
C15—C18—C21	116.4 (6)	C104—C108—O109	108.5 (4)
O19—C18—C21	104.4 (6)	C107—C108—O109	112.6 (4)
C15—C18—H181	109.7	C104—C108—H1081	112.3
O19—C18—H181	103.9	C107—C108—H1081	111.6
C21—C18—H181	116.8	O109—C108—H1081	111.1
C18—O19—C20	103.4 (5)	C108—O109—C110	112.8 (4)
C14—C20—O19	105.2 (5)	O109—C110—H1101	108.2
C14—C20—H201	112.3	O109—C110—H1102	110.3
O19—C20—H201	110.2	H1101—C110—H1102	109.7
C14—C20—H202	109.4	O109—C110—H1103	109.9
O19—C20—H202	110.7	H1101—C110—H1103	108.6
H201—C20—H202	109.0	H1102—C110—H1103	110.1
C18—C21—N22	113.0 (6)	C104—C103—O102	108.8 (4)
C18—C21—H211	108.8	C104—C103—O130	127.7 (4)
N22—C21—H211	110.2	O102—C103—O130	123.5 (5)
C18—C21—H212	106.9	C103—O102—C101	117.7 (4)
N22—C21—H212	108.2	O102—C101—C131	122.3 (5)
H211—C21—H212	109.7	O102—C101—C135	118.5 (5)
C21—N22—N23	115.5 (6)	C131—C101—C135	119.1 (5)
N22—N23—N24	170.6 (8)	C101—C131—C132	120.7 (5)
C14—O25—C26	113.6 (5)	C101—C131—F140	120.4 (5)
O25—C26—H261	108.5	C132—C131—F140	118.9 (5)
O25—C26—H262	108.8	C131—C132—C133	119.6 (5)
H261—C26—H262	109.5	C131—C132—F139	120.1 (6)
O25—C26—H263	109.7	C133—C132—F139	120.3 (5)
H261—C26—H263	110.5	C132—C133—C134	120.1 (5)
H262—C26—H263	109.8	C132—C133—F138	119.8 (5)
C4—O28—C29	115.7 (4)	C134—C133—F138	120.1 (6)
O28—C29—H291	110.5	C133—C134—C135	120.4 (6)
O28—C29—H292	109.7	C133—C134—F137	120.8 (5)
H291—C29—H292	108.3	C135—C134—F137	118.8 (6)
O28—C29—H293	109.8	C134—C135—C101	120.1 (5)
H291—C29—H293	109.7	C134—C135—F136	119.3 (5)
H292—C29—H293	108.9	C101—C135—F136	120.5 (5)
C1—C31—C32	120.6 (8)	C104—O128—C129	114.9 (4)
C1—C31—F40	119.8 (6)	O128—C129—H1291	108.6
C32—C31—F40	119.6 (7)	O128—C129—H1292	110.8
C31—C32—C33	120.0 (9)	H1291—C129—H1292	110.4
C31—C32—F39	120.1 (10)	O128—C129—H1293	109.1
C33—C32—F39	119.9 (8)	H1291—C129—H1293	109.0
C32—C33—C34	119.8 (7)	H1292—C129—H1293	108.9
C32—C33—F38	119.8 (10)	C114—O125—C126	115.0 (4)
C34—C33—F38	120.5 (9)	O125—C126—H1261	109.3
C33—C34—C35	119.7 (8)	O125—C126—H1262	110.3
C33—C34—F37	121.0 (7)	H1261—C126—H1262	108.8
C35—C34—F37	119.3 (9)	O125—C126—H1263	109.1
C1—C35—C34	121.3 (8)	H1261—C126—H1263	109.8
C1—C35—F36	119.0 (6)	H1262—C126—H1263	109.4
C34—C35—F36	119.6 (7)	C118—C221—N222	106.0 (7)
O116—C117—H1171	108.7	C118—C221—H2211	110.6
O116—C117—H1172	109.8	N222—C221—H2211	110.7
H1171—C117—H1172	110.1	C118—C221—H2212	109.4
O116—C117—H1173	108.7	N222—C221—H2212	110.5
H1171—C117—H1173	109.2	H2211—C221—H2212	109.5
H1172—C117—H1173	110.4	C221—N222—N223	114.5 (8)
C117—O116—C115	113.9 (7)	N222—N223—N224	170.8 (17)

### Hydrogen-bond geometry (Å, °)

**Table:**

*D*—H···*A*	*D*—H	H···*A*	*D*···*A*	*D*—H···*A*
C5—H52···O27^i^	0.97	2.23	3.131 (10)	153
C20—H202···N222^ii^	0.97	2.32	3.269 (10)	167
C26—H263···N222^ii^	0.97	2.51	3.214 (10)	129
C29—H293···O27^i^	0.97	2.49	3.278 (10)	139
N12—H121···O128^iii^	0.87	2.36	3.200 (10)	164
C129—H1292···O127^i^	0.96	2.46	3.370 (10)	158
C126—H1261···O27	0.96	2.47	3.202 (10)	133
N112—H1121···O28	0.86	2.33	3.147 (10)	159

Symmetry codes: (i) *x*, *y*−1, *z*; (ii) −*x*+1/2, *y*+1/2, −*z*; (iii) *x*−1/2, *y*+1/2, *z*.

### Table 2 Selected torsion angles (°) leading to extended and contracted geometries.

**Table:**

Atoms	Molecule *A*	Molecule *B*
O6—C7—C11—N12	71.6	104.2
C7—C11—N12—C13	78.1	-176.8
C11—N12—C13—C14	-165.8	-174.1
N12—C13—C14—O25	-18.6	-155.9
